# Anti-inflammatory and antioxidant properties of six common fruit extracts: An *in vitro* study

**DOI:** 10.6026/973206300211986

**Published:** 2025-07-31

**Authors:** Sujata Chhabile, Arun Dodamani, Prashanth Vishwakarma, Harish Jadav, Ankita Gadekar, Snehal Chintale

**Affiliations:** 1 Department of Public Health Dentistry, ACPM Dental College, Dhule, Maharashtra, India

**Keywords:** Oxidative stress, fruit extract, glycaemic index, anti-inflammatory, anti-oxidant, standard drug

## Abstract

Fruits like *ananas comosus*, *Malus pumila* and *Manilkara zapota* are known to have antioxidant activity *in vitro*, while *Mangifera indica* and *M. pumila* have moderate anti-inflammatory effects. Extracts were tested at 10-50 µL and compared with standard drugs. Antioxidant activity was concentration-dependent and statistically significant (p ≤ 0.05). Anti-inflammatory activity, though notable, was less than diclofenac. Thus, the potential of fruit extracts in managing oxidative stress and inflammation is reported.

## Background:

The human body relies on various defense mechanisms to maintain health and respond to external threats. One such essential mechanism is inflammation, which serves as an involuntary yet vital physiological response of the immune system to stimuli such as pathogens, toxins, or tissue injury. This response is marked by redness, swelling, pain, heat and functional loss, aiming to eliminate harmful agents and initiate healing [1]. However, chronic inflammation is a major contributor to the onset and progression of numerous diseases, including cancer, diabetes, arthritis, cardiovascular disorders and neurological conditions [2]. Therefore, effective management of inflammation is crucial in reducing the burden of chronic diseases. Oxidative stress, defined as an imbalance favoring oxidants over antioxidants, leads to molecular damage and disruption of redox signaling and control [3]. Oxidative stress and inflammation are closely interlinked, often exacerbating each other and fueling a vicious cycle that underpins the pathogenesis of many chronic diseases [4]. Oxidative stress results from excessive production of reactive oxygen species (ROS) and inadequate antioxidant defenses, leading to cellular and tissue injury [5, 6]. Chronic oral inflammation has systemic implications, as pro-inflammatory cytokines and oxidative stress mediators may enter the bloodstream and contribute to conditions such as diabetes, rheumatoid arthritis and cardiovascular disease [7]. Non-steroidal anti-inflammatory drugs (NSAIDs), such as diclofenac, are commonly used for managing inflammation by inhibiting cyclooxygenase enzymes responsible for prostaglandin synthesis [8, 9]. Likewise, synthetic antioxidants like ascorbic acid are employed to neutralize free radicals and alleviate oxidative stress. However, these agents often suffer from limitations such as poor bioavailability, degradation during storage and reduced efficacy when used alone [10]. In recent years, natural alternatives like fruits have garnered considerable interest due to their rich content of bioactive compounds with dual antioxidant and anti-inflammatory potential. Fruits contain phytochemicals such as flavonoids, polyphenols, carotenoids and vitamins that help modulate inflammatory pathways, scavenge free radicals and enhance endogenous antioxidant enzyme activity [11]. Studies have shown that fruits with high polyphenol content-such as pomegranates and berries-exhibit beneficial effects in mitigating inflammation and oxidative stress [12]. Furthermore, fruits are associated with fewer side effects than synthetic agents, making them a safer and more holistic therapeutic option [13]. Another important consideration is the glycaemic index (GI) of fruits. The GI reflects how quickly carbohydrate-rich foods raise blood glucose levels. Low-GI foods contribute to more stable blood sugar levels, potentially reducing inflammation and oxidative damage [14]. Therefore, evaluating the GI of fruits is essential in assessing their therapeutic benefits [15]. Despite the known benefits of fruits, limited research has compared their antioxidant and anti-inflammatory efficacy against standard agents such as diclofenac and ascorbic acid. Moreover, comparative evaluations of fruits with varying glycaemic indices-such as watermelon, apple, sapodilla, pineapple, mango and orange-remain scarce. Filling this gap can provide valuable insights into the therapeutic value of these fruits and inform dietary or adjunctive treatment strategies. Therefore, it is of interest to assess the anti-inflammatory and antioxidant properties of fruits categorized by glycaemic index and compare their effectiveness with diclofenac and ascorbic acid.

## Materials and Methodology:

## Study setting:

The study was conducted in the laboratory of the department of biochemistry assessing the anti-inflammatory and antioxidant properties of commonly consumed fruits: mango (*Mangifera indica*), apple (*Malus pumila*), sapodilla (*Manilkara zapota*), orange (*citrus sinensis*), pineapple (*ananas comosus*) and watermelon (citrus lanatus). Standardized protocols were followed to minimize external variability and ensure accurate results.

## Study design:

An *in vitro* study

## Sample selection:

Fruits were categorized based on their glycaemic index into high, medium and low groups. Two fruits were randomly selected from each category to evaluate their anti-inflammatory and antioxidant activity.

The six fruits included in the study were:

[1] High glycaemic index: *Citrullus lanatus*, *Mangifera indica*

[2] Moderate glycaemic index: *Manilkara zapota*, *citrus sinensis*

[3] Low glycaemic index: *ananas comosus*, *Malus pumila*

These samples were ripe fruits purchased from a grocery store. The edible parts of each fruit were used for extraction.

## Materials:

Chemicals and reagents were used, including bovine serum albumin (BSA), 1N Hydrochloric acid (HCL), Diclofenac sodium, DPPH (2,2- diphenyl-1 -picrylhydrazyl), tris HCL buffer (50 Mm, PH 7.4), ascorbic acid and distilled water to ensure accurate biochemical analysis.

## Extraction and preparation of samples:

Fruit samples were extracted using a standardized aqueous extraction method to obtain bioactive compounds from the selected fruits. Fresh ripe mango (*Mangifera indica*), apple (*Malus pumila*), sapodilla (*Manikara zapota*), orange (*Citrus sinesis*), pineapple (*Ananas cosmous*) and watermelon (*Citrullus lanatus*) were purchased from a grocery store and stored at 4°C before processing to maintain freshness [1212]. The edible portion (pulp) of each fruit was separated, ensuring the exclusion of seeds and peels unless otherwise required. (10g) of fruit pulp was weighed and crushed using a sterile mortar and pestle. The crushed pulp was mixed with 100 ml of distilled water and then stirred for 15 minutes using a glass rod to enhance solubility. The mixture was subjected to heat-assisted extraction without degrading heat-sensitive compounds. After heating, the solution was filtered first using muslin cloth and then through Whatman No.1 filter paper for finer filtration, removing solid residues and obtaining a clear extract.

## Anti-inflammatory activity using bovine serum albumin denaturation assay:

The anti-inflammatory activity of the fruit extract was evaluated using the Bovine serum albumin (BSA) denaturation assay, which measures the ability of the extract to inhibit protein denaturation, a key process in inflammation. In this assay, 0.45 mL of 1% BSA solution was mixed with 0.05 ml of fruit extract at different concentrations (10, 20, 30, 40 and 50µL). The pH of the mixture was adjusted to 6.3 using 1N hydrochloric acid and the samples were incubated at room temperature for 20 minutes. After incubation, the mixture was heated at 55°C for 30 minutes using a water bath and then allowed to cool to room temperature. The absorbance was measured at 660nm using UV-Vis spectrophotometer and diclofenac sodium was used as the standard anti-inflammatory drug for comparison. The percentage inhibition of protein denaturation was calculated using the formula:

%Inhibition = Absorbance of control- Absorbance of samplex100/Absorbance of control

## Antioxidant activity using DPPH (2, 2- diphenyl- 1- picrylhydrazyl) assay:

The antioxidant activity of fruit extract was evaluated using the DPPH (2, 2-diphenyl-1-picrylhydrazyl) free radical scavenging assay, which measures the ability of the extracts to neutralize free radicals. In this assay, fruit extracts were tested at five different concentrations (10, 20, 30, 40 and 50µL) to observe a dose-dependent response. A mixture containing 1 ml of 0.1 mm DPPH in methanol and 450µL of 50mM Tris -HCL buffer (pH 7.4) was prepared. The fruit extract was then added to the DPPH solution and the mixture was incubated at room temperature in the dark for 30 minutes to prevent auto-oxidation. After incubation, the absorbance was measured at 517 nm using a UV-Vis spectrophotometer, with ascorbic acid used as the standard antioxidant for comparison. The percentage of free radical scavenging activity was calculated using the formula.

(Absorbance of control - absorbance of the test)/Absorbance of control) x 100

## Statistical analysis:

The statistical analysis in this study was conducted to determine the significance of differences in antioxidant and anti-inflammatory activities among fruit extract at various concentrations. Mean ± standard deviation (SD) was used for descriptive statistics. One-way ANOVA was performed to compare the activities of different fruit extracts, with a significance level set at p ≤ 0.05. If significant differences were found, post hoc Tukey's test was used for pairwise comparisons between fruit extracts. To compare the effectiveness of fruit extracts with standard drugs (Ascorbic Acid for antioxidant activity and Diclofenac sodium for anti-inflammatory activity), an independent t-test was conducted. All statistical analyses were performed using SPSS version.20 IBM, USA software, ensuring accuracy and reliability in data interpretation.

## Results:

The antioxidant activity of six different fruits was evaluated at five concentration levels (10, 20, 30, 40 and 50) Table 1 (see PDF). Shows the antioxidant activity of the six fruit extracts at different concentrations. The results indicate a concentration-dependent increase in activity, with *ananas comosus*, *Malus pumila* and *Manilkara zapota* exhibiting the highest antioxidant potential. Statistical analysis using one-way ANOVA indicated significant differences in antioxidant activity at most concentration levels (p ≤ 0.05), except for the 30% concentration, which was not significantly different across fruits. Table 2 (see PDF) presents the pairwise comparison of antioxidant activity among different fruits. The results indicate that *Citrullus lanatus* had significantly lower antioxidant activity than most other fruits, particularly at lower concentrations. The antioxidant potential of, *ananas comosus*, *Manilkara zapota* and *Malus pumila* was comparable, suggesting a similar composition of active compounds. Table 3 (see PDF) shows the anti-inflammatory activity of the same six fruits was analysed across five concentrations. Unlike antioxidant activity, the differences in anti-inflammatory potential were less pronounced. At 10% concentration, statistical significance (p= 0.008) indicated variation among fruits, but at higher concentrations, differences were not statistically significant (p > 0.05). The result implies that while some fruits have superior antioxidant properties, their anti-inflammatory effects may be less distinct. Table 4 (see PDF) provides a pairwise comparison of the anti-inflammatory acts of *Mangifera indica*. At lower concentrations 10 and 20, it showed significantly different activity from other fruits, while at higher concentrations, all fruits exhibited similar anti-inflammatory potential. Table 5 (see PDF) compares the antioxidant activity of fruits with a standard, the results indicate that all fruits exhibited lower activity, with significant differences (p ≤ 0.05) at all concentrations. However, the antioxidant potential of *ananas comosus*, *Malus pumila* and *Manilkara zapota* was relatively closer to the standard, especially at higher concentrations. Table 6 (see PDF) presents the comparison of anti-inflammatory activity with the standard drug. The anti-inflammatory activity of the tested fruits was generally lower than the standard drug. While *Mangifera indica* and *Malus pumila* showed significant differences at multiple concentrations others like *Citrullus lanatus* and *ananas comosus* had fewer significant differences. These results suggest that while fruits exhibit anti-inflammatory properties, they may not match the potency of standard pharmaceutical agents.

## Discussion:

The last few decades have seen increased interest in the medicinal applications and food additive potential of naturally occurring anti-inflammatory and antioxidant agents, including herbs, spices, plants and fruits. The present study evaluated the antioxidant and anti-inflammatory activities of aqueous extracts from six commonly consumed fruits: *ananas comosus* (pineapple), *Malus pumila* (apple), *Manilkara zapota* (sapodilla), *Citrullus lanatus* (watermelon), *citrus sinensis* (orange) and *Mangifera indica* (mango). The results demonstrated variability in antioxidant capacity among the fruit extracts, with *A. comosus*, *M. pumila* and *M. zapota* showing the highest free radical scavenging activity. In contrast, *M. indica* and *M. pumila* exhibited notable but lower anti-inflammatory effects than diclofenac sodium, though they showed significance at various concentrations. The DPPH assay showed a concentration-dependent increase in antioxidant activity for all tested fruit extracts. *A. comosus* demonstrated the highest antioxidant activity, attributed to its rich content of ascorbic acid, flavonoids and polyphenols [13]. Bromelain, a proteolytic enzyme present in *A. comosus*, has known antioxidant and anti-inflammatory effects [14]. Antioxidant and cytotoxic effects of *A. comosus* extracts in breast cancer cell lines were also been seen [15]. In this study, *M. pumila* also exhibited strong antioxidant activity, aligning with findings by Sivapalan *et al.* who reported potent antioxidant properties attributed to flavonoids such as proanthocyanidin B1/B2, catechin, epicatechin, cyanidin-3-O-galactoside and quercetin derivatives [16]. Similarly, *M. zapota* contains polyphenolic compounds contributing to its antioxidant potential. *M. zapota* pulp had higher antioxidant activity than peel and seed via DPPH and β-carotene bleaching assays [17]. High ORAC activity for *M. zapota* (without peel/seed) compared to other fruits like strawberry and banana is also seen [18]. Although *C. lanatus*, *C. sinensis* and *M. indica* demonstrated antioxidant activity, it was lower than that of *A. comosus*, *M. pumila* and *M. zapota*. This may be due to differences in the concentration and bioavailability of antioxidant compounds. In this study, *C. lanatus* showed relatively lower antioxidant activity, contrasting prior findings [19]. This inconsistency may be due to variations in extraction methods, fruit ripeness, or environmental conditions. However, *C. lanatus* peel had higher antioxidant activity than its pulp, based on DPPH and ABTS assays, attributing this to differences in phytochemical composition [20]. Similarly, although *M. indica* is rich in carotenoids and polyphenols, it showed moderate antioxidant capacity, possibly due to differences in aqueous extraction efficiency [21]. Anti-inflammatory activity, evaluated using the bovine serum albumin (BSA) denaturation assay, was generally lower than that of diclofenac sodium. However, *M. indica* and *M. pumila* showed significant inhibition of protein denaturation at several concentrations, indicating noteworthy anti-inflammatory potential. *M. indica*'s effects may be attributed to compounds like mangiferin, known to inhibit key inflammatory pathways [2]. Aqueous leaf extracts of *M. indica* significantly reduced inflammation in rat models, showing inhibition comparable to diclofenac at 10 mg/kg [23]. Topical and oral administration of *M. indica* extract reduced ear edema in mice and inhibited inflammatory mediators like TNF-α and PGE2 [24, 25, 26-27]. These findings support the therapeutic potential of fruit-derived antioxidants for use in functional foods and nutraceuticals. The strong antioxidant properties of *A. comosus*, *M. pumila* and *M. zapota* highlight their value in combating oxidative stress. The moderate anti-inflammatory activity of *M. indica* and *M. pumila* underscores their promise as natural anti-inflammatory agents. Future research should isolate and quantify specific bioactive compounds using techniques like high-performance liquid chromatography (HPLC) and mass spectrometry (MS) and validate these findings in *in vivo* and clinical trials.

## Conclusion:

*A. comosus*, *M. pumila* and *M. zapota* had strong antioxidant activity *M. indica* and *M. pumila* showed notable anti-inflammatory effects. These fruits may serve as natural therapeutic agents in managing oxidative stress and inflammation. Further molecular-level studies are needed to confirm their clinical relevance.

## Sources of Support:

Nil
